# Stargazin and γ4 slow the channel opening and closing rates of GluA4 AMPA receptors

**DOI:** 10.1038/s41598-019-45870-0

**Published:** 2019-07-02

**Authors:** Vincen D. Pierce, Li Niu

**Affiliations:** Department of Chemistry, and Center for Neuroscience Research, University at Albany, SUNY, Albany, New York 12222 United States

**Keywords:** Biophysics, Kinetics

## Abstract

As auxiliary subunits, transmembrane AMPA receptor regulatory proteins (TARPs) are known to enhance macroscopic current amplitude and alter kinetic properties of AMPA receptors on slow time scale, such as desensitization rate. Whether TARPs affect the rate of AMPA channel opening and closing, however, remains elusive. Using a laser-pulse photolysis technique, we investigated the effect of γ-2 (stargazin, a type 1a TARP) and γ-4 (a type 1b TARP) on the channel-opening and channel-closing rate constants (i.e., k_op_ and k_cl_) of GluA4 homomeric channels. We found both TARPs slow the k_op_ and k_cl_ by 4-fold and 3-fold, respectively, without appreciable change of channel-opening probability, as compared with GluA4 channel alone. On the other hand, γ-4 has a stronger effect on slowing the channel desensitization rate than γ-2; yet, γ-2 causes a much more pronounced left shift of the dose-response relationship by increasing its affinity towards glutamate than γ-4. Our study shows that on the faster time scale, the major impact of TARP association with GluA4 is to lengthen the lifetime of the open channel, which is slow to form, to allow a larger charge transfer through the open channel that closes more slowly, without appreciable change of channel opening probability.

## Introduction

AMPA receptors are a subtype of glutamate ion channels. AMPA receptors mediate the majority of the fast neurotransmission in the mammalian central nervous system (CNS), and they are critical for expression of plasticity^1,2^. AMPA receptors are tetramers, assembled from one or more pore-forming subunits, GluA1-4^1,2^. *In vivo*, AMPA receptor activities are modulated by a group of proteins known as transmembrane AMPA receptor regulatory proteins (TARPs)^3–7^. TARPs consist of eight gene products, and they are categorized into two groups: type I TARPs that comprise γ-2 (or stargazin), γ-3, γ-4, and γ-8, and type II TARPs that include γ-5 and γ-7^8,9^. Type I and Type II TARPs have unique sequences^10,11^. For example, all type I TARPs have class I PDZ binding motif at the end of their C-termini^10^. All type I TARPs have similar effects on facilitating AMPA receptor trafficking^12^, synaptic enrichment, receptor targeting^13^ and recycling^14^. At the receptor level, type I TARPs slow the rate of desensitization, deactivation and miniature excitatory postsynaptic current (mEPSC) decay. Type I TARPs also reduce polyamine block^15,16^, but increase the conductance of AMPA channels^17–19^. Type I TARPs are further divided into Type Ia (γ-2 and γ-3) and Type Ib (γ-4 and γ-8) on the basis of their differential modulations of AMPA receptor gating and pharmacological properties^19–21^. Extensive studies of stargazin or γ-2 have largely contributed to the current understanding of how TARPs modulate AMPA receptor activities^15,16,22–28^.

One of the most significant roles of TARPs is that TARPs potentiate macroscopic current amplitude of AMPA receptors. Current amplitude is related to current rise time and channel activation process. The rise time is dependent on the magnitude of both channel-opening (k_op_) and channel-closing (k_cl_) rate constants^29^. To date, however, whether TAPRs affect k_op_ and/or k_cl_ remains poorly understood. This is largely due to the fact that AMPA receptors open their channels upon binding of glutamate in the microsecond (μs) time region but channels become desensitized even in the millisecond (ms) time domain. Yet traditional kinetic approaches, such as fast solution exchange techniques, which are routinely used, are not fast enough for measuring the rate of AMPA receptor channel opening^29,30^. Several hypotheses have been nonetheless proposed to account for the effect of TARPs on enhancing macroscopic current amplitude. For example, potentiation of the current amplitude is thought to be a result of a faster rate of channel opening when TARPs, such as γ-2, are bound to AMPA receptors – this is a hypothesis formed largely from measurements on a slower time scale, i.e. deactivation and desensitization rates^16^. The primary role of γ-2 has been postulated to increase the rate of channel opening to explain that γ-2 slows deactivation but does not alter the mean duration of channel openings^16^. Milstein *et al*. reported^9^ that γ-2 and γ-4 each slowed the rise time of GluA1 channels. The interpretation of a slower current rise, however, is based on model fitting of the data involving channel desensitization^9^. Furthermore, Zhang *et al*.^19^ have proposed that the channel-opening probability (P_open_) of an AMPA receptor is increased in the presence of TARPs, whereas Soto *et al*.^15^ found the P_open_ is unchanged when γ-2 is complexed with GluA4. Given P_open_ can be expressed from both k_op_ and k_cl_^29^, it is important to determine k_op_ and k_cl_ of an AMPA receptor in the presence of TARPs. Whether TARPs affect the rate of AMPA receptor channel opening is a fundamental question in understanding the functional role of TARPs. In this study, we ask how γ-2, and separately γ-4, modulates the channel-opening rate of GluA4. It should be especially noted that γ-2 is a type Ia TARP, while γ-4 is a type Ib TARP. Our results are therefore expected to further show any functional difference between the two TARPs in modulating the channel-opening kinetics, because the same AMPA receptor, i.e., GluA4, is used in our study.

To measure the rate of GluA4 channel opening, we use a laser-pulse photolysis technique, combined with whole-cell current recording. By this technique, glutamate is generated photolytically from “caged glutamate” or γ-*O*-(α-carboxy-2-nitrobenzyl)glutamate with a time constant of ~30 μs^30,31^. Using this technique has previously enabled us to characterize the kinetic mechanism of glutamate-induced channel opening of GluA4 homomeric channels^30^. In the laser-pulse photolysis measurement, we have shown that the time course of channel opening in the μs time region can be cleanly separated from the channel desensitization reaction that occurs on the ms time scale. Therefore, our measurement of k_op_ and k_cl_ is independent of channel desensitization reaction or the fitting that involves channel desensitization rate parameters^30^. As such, the use of this rapid kinetic technique enables us to investigate whether γ-2 or γ-4 affects k_op_ and/or k_cl_, and if so, whether γ-2 and γ-4 differentially modulate the channel-opening kinetic mechanism of GluA4. Whether potentiation of the macroscopic current amplitude necessarily involves an increase in P_open_ can be further addressed.

## Results

### Experimental design

To test our hypothesis by which TARPs affect k_op_ and k_cl_, and different TARPs affect these rate constants differently, we designed our study with the use of a single AMPA receptor type but with two different TARPs. Specifically, we measured the effect of γ-2 and separately γ-4 on the channel-opening rate process of GluA4 receptors. In our experiments, we transiently expressed GluA4 in embryonic human kidney (HEK-293) cells, because GluA4 is known to form functional, homomeric channels when expressed in a heterologous host system, such as HEK-293 cells^30^.

*In vivo*, GluA4 is transiently expressed in pyramidal cells during synapse formation and reorganization^32,33^. These GluA4-containing, early formed synapses are dynamic and very susceptible to activity-dependent regulation^34^. In addition, a recent study of the postmortem Alzheimer’s disease cortex shows profound reductions of NPTX2 and GluA4^35^. GluA4 is also one subunit originally identified to directly interact with γ-2^12^. That said, the main rationale of our experimental design is to investigate whether γ-2 and γ-4 affect the channel-opening process of GluA4, and if so, whether γ-2 and γ-4 show any functional difference in modulating the channel-opening kinetics of the same receptor.

### Expression of TARP-containing channels in HEK-293 cells

**Figure 1:**
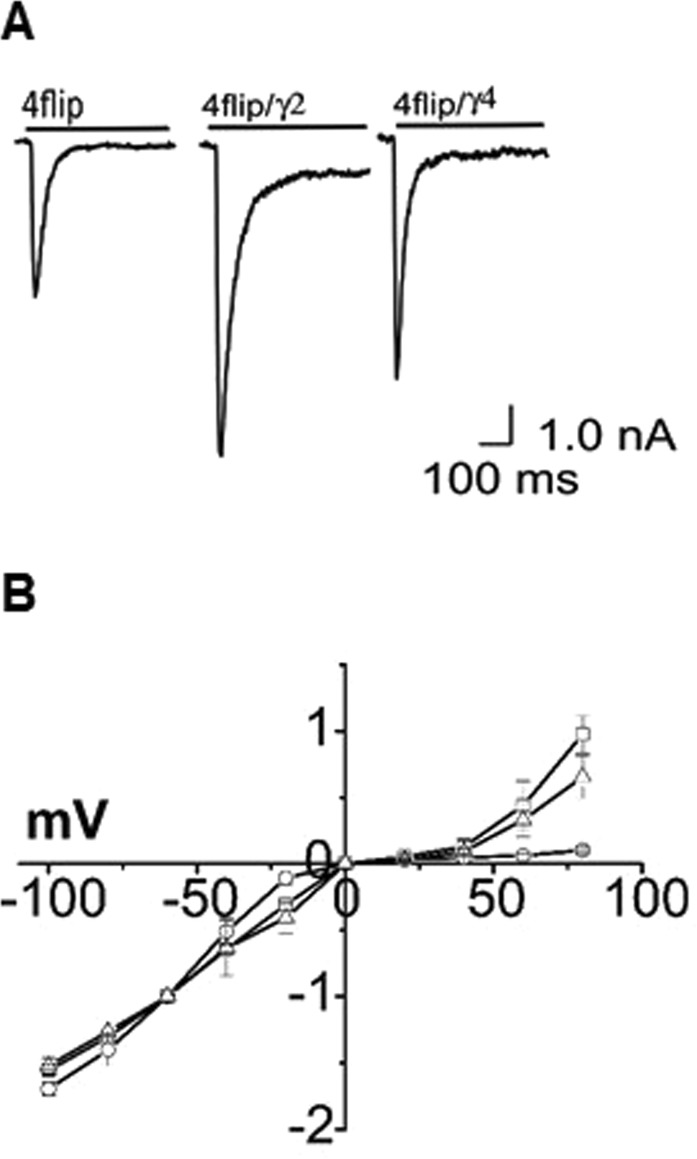
Representative whole-cell current response and I-V relationship. (**A**) Representative whole-cell current response of GluA4, GluA4/γ2, and GluA4/γ4, as labeled, to 500 µM glutamate. (**B**) I-V relationships for GluA4 (o), GluA4/γ2 (□), and GluA4/γ4 (Δ). Whole-cell current was measured from HEK-293 cells voltage clamped from −100 mV to +80 mV. These cells expressed each of the receptor of interest. In each receptor measurement, the data were collected from ~15 cells and normalized to the current amplitude collected at −60 mV.

First, we wanted to demonstrate that GluA4 homomeric channels and a TARP could be co-expressed in HEK-293 cells. We also repeated some of the experiments reported in literature to show we could consistently observe the same channel properties. To begin, we transiently expressed GluA4 in HEK-293 cells with and without a TARP. When either γ-2 or γ-4 was co-expressed, the current amplitude mediated by homomeric GluA4 channels was higher (Fig. 1A), as expected^18^. To verify the formation of GluA4/TARP complexes in HEK-293 cells, we further measured their current-voltage (I-V) relationships. GluA4 homomeric channels lacking a TARP displayed an inwardly rectifying I-V curve, whereas an I-V relationship for TARP-bound GluA4 channels was trending towards linearity (Fig. 1B). The I-V relationships we observed are similar to those reported earlier^15,23,36,37^.

An inward rectification in I-V curve, as observed in AMPA receptor alone or without TARP, is attributed to intracellular polyamine block of the receptor^15^. Thus, the reduction, but not elimination, of rectification reflects a TARP-mediated attenuation of intracellular polyamine blockade^15^. In fact, a study by Soto *et al*.^36^ showed that the proximal region of the C-terminus of γ-2 is important for attenuating polyamine block. The same study also showed that γ-2 increases the channel permeability, rather than the pore size, of homomeric AMPA receptors^36^. It should be further noted that the relative amount of the plasmid of a TARP used in our study (see Methods) was similar to those previously reported^22,23,38^. Doubling the plasmid amount of either γ-2 or γ-4 as compared with the plasmid amount of GluA4 did not further change the profile of the I-V curve nor the channel desensitization rate constant.

### Effect of γ-2 and γ-4 on the GluA4 channel desensitization rate

**Figure 2:**
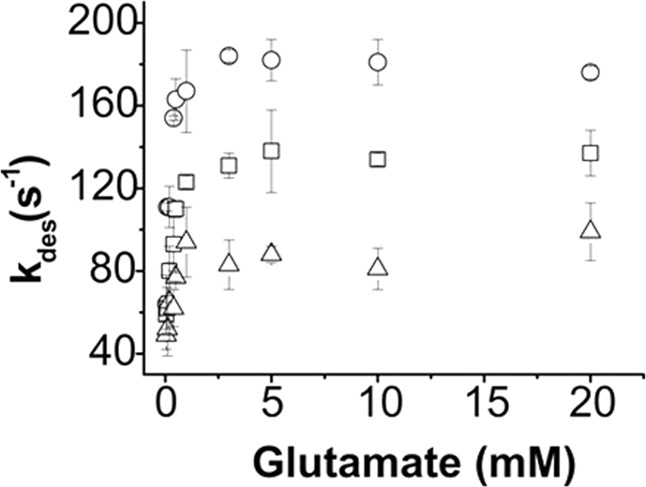
Dependence of the desensitization rate constant, k_des_, on glutamate concentration. The k_des_ values are shown for GluA4 (o), GluA4/γ2 (□) and GluA4/γ4 (Δ). Each point is an average of at least three measurements from three cells. The desensitization rate constant is shown with the standard error of the mean.

As shown (Fig. 1A), the whole-cell current response of GluA4 to glutamate increased initially due to channel opening, but quickly fell back as the channel became desensitized in the continued presence of glutamate. The desensitization followed a first-order rate (>98%) in the absence and presence of a TARP, and in the entire range of glutamate concentrations (Fig. 2).

As seen in Fig. 2, GluA4 channel desensitized faster as glutamate concentration became higher^30^. When a TARP was present, however, the desensitization rate constant (k_des_) of GluA4 became smaller or channel desensitization became slower (Fig. 2). Our observation was similar to an earlier report of GluA4 with the same TARPs, namely γ-2 and γ-4, although the absolute magnitude of the maximum k_des_ we determined is lower than the corresponding one in the early study^39^. In addition, γ-4 was previously shown to slow more significantly the channel desensitization rate than γ-2, not just on GluA4 but also on other AMPA receptors^39,40^. It should be noted that k_des_ values at various glutamate concentrations were invariant when a TARP was co-transfected under the condition we used (see Methods). Furthermore, the apparent reduction of channel desensitization rate by either γ-2 or γ-4 was dependent on agonist concentration, but plateaued around 5 mM glutamate concentration (Fig. 2). The maximum rate constant for GluA4 channel desensitization for each of the three channel types is summarized in Table 1. In addition, both γ-2 and γ-4 further affected the extent of desensitization. As seen in Fig. 1A, both γ-2 and γ-4 reduced the extent of desensitization, namely the percentage of the steady-state current response, albeit γ-2 showed a stronger effect on the reduction of the extent of desensitization. Our observation is qualitatively similar to the one reported earlier^40^.

**Table 1:** Summary of constants of GluA4, GluA4/γ2 and GluA4/γ4.

Receptor	k_des_ (s^−1^)	EC_50_ (mM)	K_1_ (mM)	k_op_ (x10^4^ s^−1^)	k_cl_ (x 10^3^ s^−1^)
GluA4	181 ± 7	0.81 ± 0.04	1.21 ± 1.42	6.41 ± 0.44	3.39 ± 0.11
GluA4/γ2	135 ± 11	0.39 ± 0.03	0.42 ± 0.49	1.32 ± 0.12	1.17 ± 0.11
GluA4/γ4	90 ± 12	0.65 ± 0.08	0.77 ± 1.31	1.70 ± 0.15	1.08 ± 0.05

Footnotes: (a) The k_des_ values are means (±SD) of those obtained from the saturated whole-cell responses evoked by 10 and 20 mM glutamate. (b) K_1_, k_op_, k_cl_, and EC_50_ values (±SEM) were yielded through fitting (see Results and Methods).

### Differential effect of γ-2 and γ-4 on the GluA4 dose-response relationship

**Figure 3:**
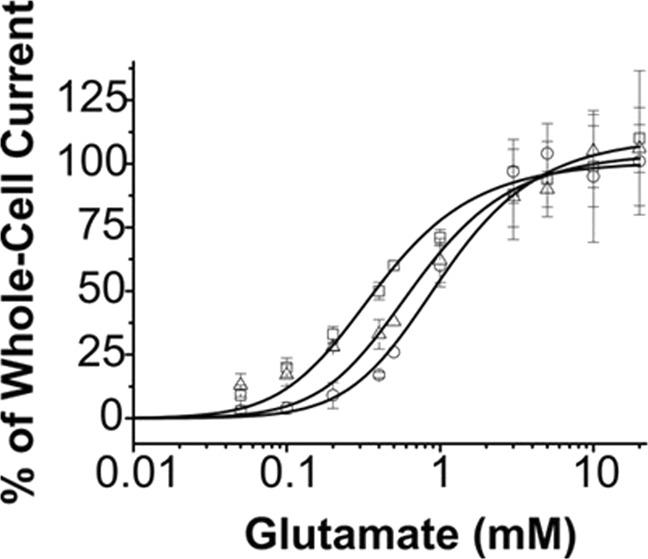
Dose-response relationship. In collecting whole-cell current responses (see representative traces in Fig. 1A), 0.5 mM glutamate was used as the control. The current amplitudes from different cells were normalized to the one obtained at 0.5 mM glutamate. The peak amplitude of a whole-cell current trace was corrected for desensitization (Methods), and the corrected current amplitude was used for dose-response plot. The average of the current amplitudes at 5, 10, and 20 mM was set to 100% for each dose-response relationship. The dose-response relationship for GluA4 (o), GluA4/γ2 (□) and GluA4/γ4 (Δ) was analyzed by nonlinear regression using Eq. 1, and separately by the Hill equation. The best-fitted parameters are: for GluA4, K_1_ = 1.21 ± 1.42 mM, Φ = 0.36 ± 0.88, I_M_R_M_ = 150 ± 104, with EC_50_ = 0.81 ± 0.04 mM; for GluA4/γ2, K_1_ = 0.42 ± 0.49 mM, Φ = 0.41 ± 0.95, I_M_R_M_ = 142 ± 99, with EC_50_ = 0.39 ± 0.03 mM; and for GluA4/γ4: K_1_ = 0.77 ± 1.31 mM, Φ = 0.39 ± 1.36, I_M_R_M_ = 145 ± 149, with EC_50_ = 0.65 ± 0.08 mM, respectively. For the Hill coefficients and various n values (i.e., the number of ligand molecules that are bound to and open a channel), see Supplemental Materials.

Both γ-2 and γ-4 led to left shift the corresponding dose-response curve (Fig. 3). However, γ-2 had a stronger effect on the dose-response curve than γ-4 (Fig. 3). The best fit of the dose-response relationship of GluA4 alone, using Eq. 1 (in Methods), yielded K_1_ = 1.10 ± 0.81 mM (solid symbol) when n = 2; n is the number of ligand molecules that bind to the receptor leading to the opening of the channel (Fig. 3). This value was consistent with the one we reported previously^29,30^. For GluA4/γ2 and GluA4/γ4 channels, K_1_ of 0.42 ± 0.49 mM and K_1_ = 0.77 ± 1.31 mM were obtained through non-linear regression, respectively. When Hill equation^41^ was used for fitting, we obtained EC_50_ value for GluA4, GluA4/γ2 and GluA4/γ4 to be 0.81 ± 0.04 mM, 0.39 ± 0.03 and 0.65 ± 0.08 mM, respectively (these and other fitted parameters are listed in the figure legend of Fig. 3, and Table 1; the Hill coefficients are provided in Table S1 in Supplemental Materials).

That both γ-2 and γ-4 caused their respective dose-response curves to left shift suggested that co-expression of either one of these TARPs would contribute to enhancing whole-cell current response of GluA4 at the same glutamate concentration (Fig. 1A), provided that the glutamate concentration is not saturating. In this aspect, our data are consistent with earlier reports about the effect of type I TARPs on potentiating AMPA receptor activities^3^. In particular, our data are qualitatively similar to those reported by Kott *et al*.^42^ in which γ-2 was found to be more potent than γ-4 in potentiating GluA4 channel-mediated current, although the conclusion from Kott *et al*. was based on oocyte current measurements. We are not, however, aware of any previous report on the determination of glutamate EC_50_ value for either GluA4/γ-2 or GluA/γ-4. Given these EC_50_ values we determined (Table 1), at a lower but the same glutamate concentration (e.g., 500 µM; Fig. 1A), it would be expected that co-expression of γ-2 would potentiate the GluA4 more strongly than γ-4; in other words, a higher current response to the same glutamate concentration would be expected from GluA4/γ-2 than from GluA4/γ-4 channels (Fig. 1A).

It should be noted that EC_50_ and K_1_ values are numerically similar (see Table 1). Unlike EC_50_, however, K_1_ came from the analysis of the dose-response relationship by the use of a minimal, general model of channel opening (see the model and Eq. 1 in Methods). Since K_1_ is the intrinsic equilibrium dissociation constant, the fact that K_1_ was affected when a TARP was present suggested interaction of either γ-2 or γ-4 affected the ligand binding affinity for GluA4/γ-2 and GluA4/γ-4. Furthermore, γ-2 seemed to have a greater effect on ligand binding affinity than γ-4 on the same receptor (Table 1).

### Differential effect of γ-2 and γ-4 on the rate of channel opening of GluA4 channels

**Figure 4:**
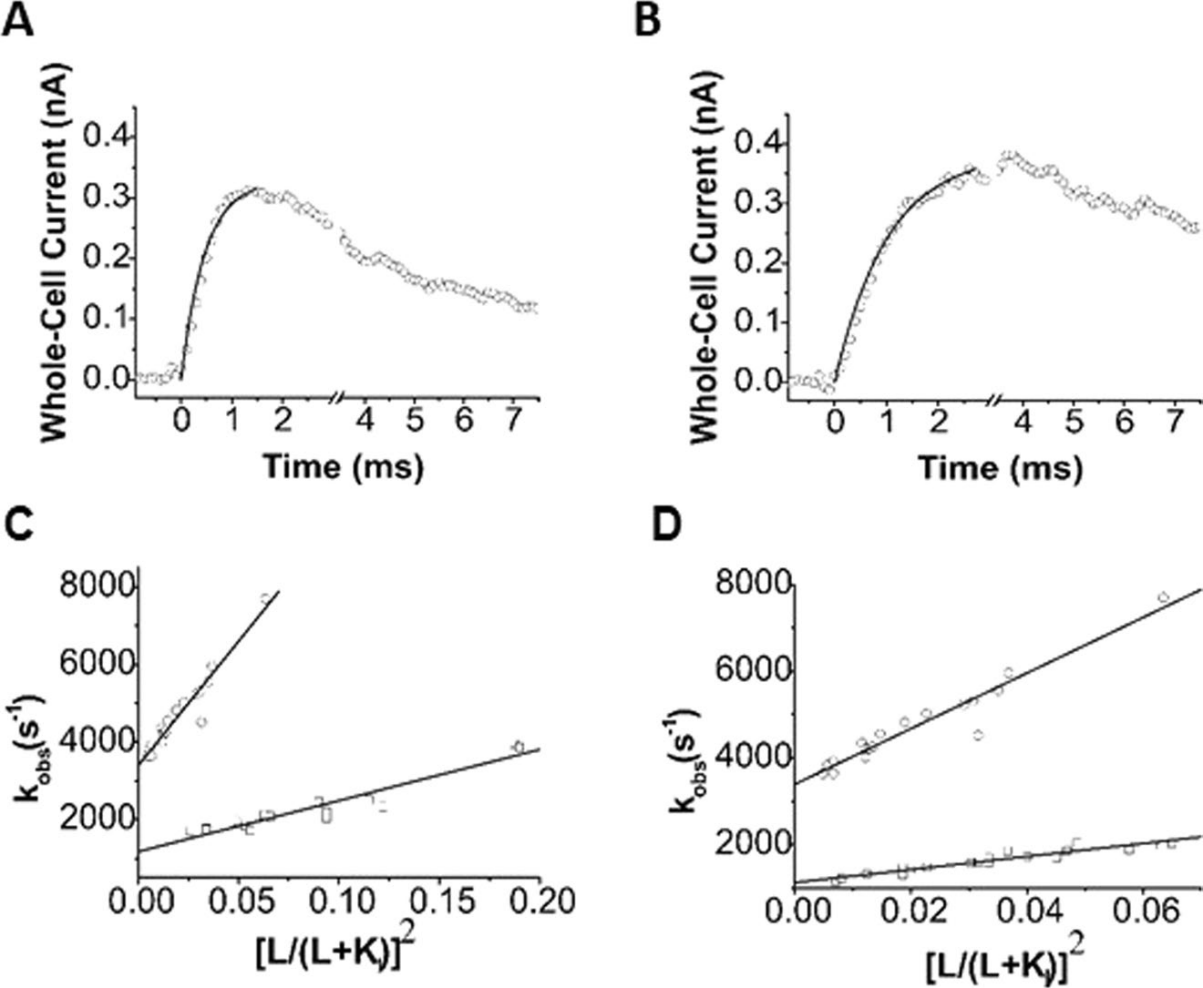
Laser-pulse photolysis measurement. (**A**) Shown here is a representative whole-cell current trace from the opening of GluA4 channel initiated by a laser-pulse photolysis of caged glutamate at time zero. For the clarity of illustration, the number of data points in the rising phase of the current were reduced. The k_obs_ of 4,213 s^−1^ was determined by fitting the rising phase to a single-exponential rate expression (solid line) using Eq. 2. (**B**) Similarly shown is a representative whole-cell current trace from the laser-pulse photolysis experiment with an HEK-293 cell expressing GluA4/γ4 channel. From this trace, k_obs_ of 1,856 s^−1^ was calculated by fitting the rising phase to a single first-order rate expression (solid line). (**C**) The linear fit of k_obs_ as a function of glutamate concentration using Eq. 3 for GluA4/γ2 (□), where GluA4 (o) is shown for comparison. The values of k_op_ and k_cl_ were determined to be 6.41 ± 0.44 × 10^4^ s^−1^ and 3.39 ± 0.11 × 10^3^ s^−1^ for GluA4, and 1.32 ± 0.12 × 10^4^ s^−1^ and 1.17 ± 0.11 × 10^3^ s^−1^ for GluA4/γ2, respectively. (**D**) Similarly, k_op_ and k_cl_ for GluA4/γ4 (□) were determined to be 1.66 ± 0.14 × 10^4^ s^−1^ and 1.06 ± 0.05 × 10^3^ s^−1^, respectively. The k_op_ and k_cl_ values for GluA4 (o) are the same as in *C*, and again are shown for comparison. The K_1_ values used for fitting for the three receptor channels are listed in Table 1. These values are similar to those determined from a nonlinear regression of the same data. The detailed nonlinear fitting results are shown in Supplemental tables.

Using the laser-pulse photolysis technique combined with whole-cell recording, we were able to measure the rate of GluA4 channel opening and therefore investigated whether γ-2 and γ-4 affected the rate of the channel opening. As shown (Fig. 4A), the rise of whole-cell current response to glutamate that was photolytically liberated at time zero followed a first-order rate process (>95%). The observed first-order rate constant or k_obs_ was determined using Eq. 2. Using Eq. 3, which was derived from a minimal model of channel opening (Methods), we further determined k_op_ and k_cl_ from the best fit of k_obs_ as a function of the concentration of photolytically released glutamate for each of the three channels. Specifically, k_op_ and k_cl_ are (6.4 ± 0.4) × 10^4^ s^−1^ and (3.4 ± 0.1) × 10^3^ s^−1^ for GluA4, (1.3 ± 0.1) × 10^4^ s^−1^ and (1.2 ± 0.1) × 10^3^ s^−1^ for GluA4/γ2, and (1.7 ± 0.2) × 10^4^ s^−1^ and (1.1 ± 0.1) × 10^3^ s^−1^ for GluA4/γ4, respectively (these constants are summarized in Table 1 as well). It should be noted that k_op_ and k_cl_ values we determined here for GluA4 homomeric channel alone or without either γ-2 or γ-4 are in good agreement with those values we published earlier^30^.

Based on all of the data we have collected from the current study, we can draw the following conclusions. First, GluA4/γ-2 and GluA4/γ-4 both have significantly slower rates of channel opening and closing, as compared with the respective rates of GluA4 homomeric channels. Specifically, γ-2 and γ-4 slow down the rate of the channel opening and closing by ~4-fold and 3-fold, respectively (Table 1). Second, there is no significant difference in either k_op_ or k_cl_ between GluA4/γ-2 and GluA4/γ-4 (Table 1). In other words, γ-2 and γ-4 modulate the rate of channel opening and channel closing of GluA4 to a similar extent (Table 1).

In estimating both k_op_ and k_cl_ for GluA4/γ-2 and GluA4/γ-4 channels, we also used nonlinear regression to fit k_obs_ as a function of glutamate concentration by Eq. 3 (all nonlinear regression fits are provided Tables S2–S5). Specifically, we took the following steps in our fitting. First, k_cl_ was estimated at a low glutamate concentration, given that, when L «  K_1_, Eq. 3 is reduced to k_cl_ ≈ k_obs_. Previously, we have determined that at ~4% of the fraction of the open channels, which corresponds to ~100 µM glutamate concentration for GluA4, k_obs_ ≈ k_cl_^29^. By this rationale, the value of k_cl_ would be independent of other parameters in Eq. 3. Furthermore, we constrained n to be integer or n = 1–4, based on the assumption that the binding of a fractional ligand molecule within 1–4 or outside of this ligand occupancy range would be incompatible with tetrameric channel configuration. Using these constraints enabled us to better estimate k_op_ and K_1_ values. We found these k_op_ and k_cl_ values, estimated in various approaches, are in good agreement (Fig. 4 and Table S5). The K_1_ value which we estimated from the rate data for either  GluA4/γ-2 or GluA4/γ-4, respectively (Tables S2B and S3B), was similar to the one we estimated from the dose-response data (Fig. 3), despite that these measurements and data analysis were independent of each other. Furthermore, the best fit of n was 2, regardless channel types (Tables S2A, S3A, and S4).

## Discussion

Our study has revealed a major finding that γ-2 or γ-4, when bound to GluA4, slows both the rate of GluA4 channel opening and channel closing by ~4-fold and 3-fold, respectively (Table 1). There does not appear to be any difference between γ-2 and γ-4 in their effects on either k_op_ or k_cl_. On a longer time scale, our data confirm that both TARPs slow the rate of channel desensitization. However, γ-4 decreases the rate of channel desensitization to half, whereas γ-2 only decreases the rate by 25% (Table 1); this observation is qualitatively consistent with an early study^40^. Furthermore, γ-2 left-shifts the dose-response curve significantly, whereas γ-4 has minimal effect on dose-response relationship. As such, GluA4/γ-2 has a K_1_ value (or EC_50_ value as well) about half of the value for GluA4, whereas GluA4/γ-4 has just a slightly smaller K_1_ (or EC_50_ value), as compared with GluA4 channel alone.

The similarities and differences in the effect of γ-2 or γ-4 on GluA4 channel properties, as summarized above, may reflect a general feature of the properties defined by type Ia TARP (e.g., γ-2) and type Ib TARP (e.g., γ-4), since these parameters are determined on the same pore-forming subunit GluA4. Earlier studies show that TARPs modulate the channel properties of AMPA receptors in a TARP subtype-dependent manner. Specifically, different type I TARPs affect deactivation and desensitization kinetics  differently^18^. For example, γ-4 and γ-8 slow the deactivation rate to a greater extent, as compared with γ-2 or γ-3^8,9^. We show γ-2 and γ-4 affect differentially EC_50_ value, but affect equally the rate of the channel-opening and channel-closing processes. Whether our results on k_op_ and k_cl_ are indicative of general properties or GluA4-specific properties awaits further studies with more TARPs and additional AMPA receptor subunits. Earlier studies of various AMPA receptor homomeric channels, such as GluA1 vs. GluA4, have indeed demonstrated that these pore-forming subunits are different in single channel properties^16,25,43,44^ and in properties of ensemble average, such as k_op_ and k_cl_ values^30^.

Our observation of a smaller k_op_ or a slower channel-opening rate, which contributes to a slower rise time, does not support an earlier prediction that potentiation of the current amplitude is a result of a faster rate of channel opening when TARPs such as γ-2 are bound to AMPA receptors, a conclusion drawn largely from measurements on a slower time scale, i.e. deactivation and desensitization rates^16^. Specifically, the primary role of γ-2 has been postulated to speed up the rate of channel opening in order to explain that γ-2 slows deactivation but without altering the mean duration of channel openings^16^. However, our finding that γ-2 and γ-4 significantly slow the rates of both channel opening and channel closing suggests that the functional impact of TARPs on a shorter time scale (i.e., during the current rise) is to lengthen the time course of channel opening. As such, more ions would pass through the open channel that now closes more slowly or stays open longer than GluA4 channel without any TARP, thereby generating a larger charge transfer through the open channel or a higher amplitude of whole-cell current.

In using a whole-cell current trace as an example, the observed k_op_ term (k_op_′) is ligand concentration dependent or k_op_′ = k_op_ [L/(L + K_1_)]^n^ as in Eq. 3. In this sense, k_obs_ = k_cl_ + k_op_′. This relationship indicates that the time course of a whole-cell current rise is a sum of the two rates. As such, the integrated current over the rising phase, which reflects the total charge passing through the open channel, is dependent on ligand concentration. We can therefore derive the following rationale. (a) To potentiate the whole-cell current amplitude, a smaller k_op_ with a TARP bound to an AMPA channel, as compared with the k_op_ without TARP, would require a smaller, or at least the same, k_cl_ without sacrificing channel-opening probability (P_open_) (see additional discussion about P_open_ below). Otherwise, reducing P_open_ would be counterproductive to the action of TARPs. (b) A smaller k_op_ in the presence of a TARP is compensated by a decrease of K_1_ value such that at the same glutamate concentration (not saturating), a higher percentage of AMPA receptors open their channels when bound with TARPs. This can be seen by the fact that K_1_ (GluA4/γ-2) < K_1_ (GluA4/γ-4) < K_1_ (GluA4); therefore, k_op_ is compensated by a factor of [L/(L + K_1_)]^n^. In other words, when TARPs are bound, opening channels less rapidly is compensated by opening more channels. Therefore, a smaller k_op_ is not inconsistent with a mechanism of potentiation by which channels open slowly but more channels open, and these channels stay open longer so that more charges can be passed, resulting a higher whole-cell current amplitude. For example, GluA4/γ-2 and GluA4/γ-4 channels have similar k_op_ and k_cl_; both are smaller than the respective values of GluA4 (Table 1). However, because GluA4/γ-2 has a lower K_1_ value than GluA4/γ-4, γ-2 induces a higher potentiation than γ-4 at the same glutamate concentration (Fig. 1A).

It should be also noted that desensitization rate is not involved, at least not appreciably, in an apparent TARP-induced potentiation of current response. If it were, that GluA4/γ-2 has a faster k_des_ as compared with GluA4/γ-4 (Fig. 2 and Table 1) would suggest that γ-2 induces a smaller potentiation as compared with γ-4. Our data (Fig. 1A) do not support this notion. Not surprisingly, our data are consistent with the notion that a higher peak amplitude in whole-cell current response to glutamate when a TARP is present should be therefore ascribed to the effect of that TARP on the rising phase or the channel-opening rate process, rather than the falling phase or the desensitization phase.

From a dynamic point of view, that the k_op_ is smaller or the rate of channel opening is slower, when either γ-2 or γ-4 is co-expressed with GluA4, and the comparison of the ratios of k_cl_ to k_op_ with and without a TARP (see Table 1) suggest that the interaction of TARPs with the pore-forming subunits stabilizes the closed-channel state, rather than destabilizes it as previously suggested^37^. In other words, that the rate of channel opening is slower is indicative of the binding of a TARP with the closed-channel state of GluA4, and the interaction between TARPs and pore-forming AMPA receptor subunits is now energetically less favorable for the AMPA channels bound with TARPs to open. Using transition state theory argument, i.e., *ΔΔG* = R T ln(k′/k (k′ is the rate for GluA4/TARP, whereas *k* is the rate for GluA4; all rate constants are from Table 1), we estimate the additional barrier height to be ~860 cal/mol for the channel-opening reaction (forward reaction) or between the initial state (i.e., closed-channel state) and the transition state. Similarly, the additional barrier height would be ~650 cal/mol for the channel-closing reaction (reverse reaction) or between the final state (open-channel state) and the transition state (assuming room temperature). In other words, once GluA4 is bound with a TARP, there is an additional 860 cal/mol energy to overcome for the channel to open; likewise, there is an additional 650 cal/mol energy to overcome for the open channel to close.

We speculate that the extra energetic requirement for the slower rate to open the channel and close it subsequently may be explained by a model previously proposed by Zhao *et al*.^27^ and Twomey *et al*.^28^. The model has been established from cryo-electronic microscopy studies of the homomeric GluA2 channel bound with γ-2 through its transmembrane domain and extracellular loops that reach to the ligand-binding domain (LBD) of the receptors. The γ-2 subunits are positioned underneath the two-fold symmetric LBD, like partially opened palms^27,28^. The extracellular loop region in γ-2 that is composed of a number of negatively charged residues interacts with the receptor LBD lower lobe, which contains a number of positively charged residues, thereby forming an electrostatic “patch”. During channel activation, γ-2 “palms” engage with LBD to stabilize intra-dimer and inter-dimer interfaces. In fact, mutating a set of highly conserved, positively charged amino acid residues on GluA2 but within this electrostatic patch almost eliminated the effects of γ-2 on GluA2 channel function^26^. Based on this model and our data, i.e., a smaller k_op_, the electrostatic interaction between γ-2 and AMPA receptor that stabilizes the closed-channel state must be disrupted before the channel can open. Conversely, when the open channel returns to the closed-state, such an electrostatic interaction must be restored. In other words, the opening and closing of the AMPA receptor channels bound with TARPs must involve the breakup and restoration of this electrostatic interaction between TARP and receptor subunits. Besides this electrostatic patch, the interaction between a TARP such as γ-2 and the C-terminal domain of the receptor could also contribute to the stabilization of the closed- and the open-channel states^37^. Although our data indicate that both the channel-opening and the channel-closing rates are slowed by either γ-2 or γ-4 interacting with GluA4, the critical effect of a TARP on GluA4 is to slow the rate of channel closing. In so doing, a TARP would enable the lifetime of the channel opening to be prolonged so that a larger charge can be transmitted through the channel that remains open in a longer time period, as compared with GluA4 channel without TARP. Otherwise, from a simple kinetic point of view, if the channel bound with a TARP opened with a slower rate but closed with the same rate as GluA4, a smaller charge transfer or a lower macroscopic current amplitude would be expected.

Given that a TARP slows both k_op_ and k_cl_ for GluA4/TARP channels as compared with GluA4 channel alone, would the interaction of a TARP cause the reduction of P_open_? To answer this question, we estimated P_open_ by using k_op_ and k_cl_ values, i.e. P_open_ = k_op_/(k_op_ + k_cl_)^30^. Because both γ-2 and γ-4 decrease k_op_ and k_cl_ roughly equally, the P_open_ should be similar. Thus, P_open_ for both γ-2 and γ-4 is estimated to be ~0.93. Furthermore, the P_open_ value for γ-2 and γ-4 is similar to P_open_ of 0.96 for GluA4 alone^30^. These results suggest that P_open_ for GluA4 bound with either TARP remains virtually unaffected, despite that both k_op_ and k_cl_ are significantly smaller, as compared with the GluA4 channels alone.

Our estimate that P_open_ value for GluA4/TARP channels is similar to P_open_ of GluA4 alone suggests TARP’s potentiation of GluA4-mediated macroscopic current amplitude does not require an increase or even a change in P_open_. This is reasonable because P_open_ is already near unity. A P_open_ of close to unity indicates that the channel opening, followed by the binding of glutamate to the receptor, is already a highly favored reaction. Furthermore, maintaining a high P_open_ or a highly favorable channel-opening reaction actually requires that the channel-closing rate be slow. If k_op_ for either GluA4/γ-2 or GluA4/γ-4 is smaller the way they are (as in Table 1) but k_cl_ were not slowed or remained the same as GluA4 alone, P_open_ would be reduced to <0.8. This analysis, again, shows the critical role of a TARP in slowing the rate of channel closing is not just to permit a longer open time for a larger ionic flux to occur but to maintain the same favorable forward reaction to open the channel to begin with. As such, the kinetic commitment to opening the channel once bound to glutamate would not be reduced by the interaction of TARPs with pore-forming subunits.

Given our data, those less frequent transitions between distinct gating modes, as seen in a single-channel study of AMPA receptor-TARP fusion proteins, are less likely due to a change of P_open_ from low to high^19^. On the other hand, our conclusion is consistent with the one from Soto *et al*.^15^ who reported that the P_open_ is unchanged when γ-2 is complexed with GluA4. However, Soto *et al*. reported a P_open_ of 0.61, which is much smaller than our value. The P_open_ value from Soto *et al*.^15^ is based on the analysis of the peak-scaled non-stationary fluctuation of the ensemble variance of all successive pairs of current responses. Our data, however, are based on the rate constant of k_cl_ and k_op_, measured directly from the time course of current rise, absent from any appreciable current desensitization. Separately, Zhang *et al*.^19^ have also reported that the channel-opening probability at the peak of the ensemble average is nearly 0.9.

We note that the k_op_ and k_cl_ values we have determined in this study are based on ensemble average of GluA4/TARP channels. As such, these rate constants do not reflect the channel activity associated with single channels with distinct conductance levels and/or kinetically distinct open-channel states, as observed in single channel recording experiments^16,19,23,25^. The rate constants we determined, for instance, are similar to those used to describe channel desensitization and deactivation, both of which are ensemble processes. That said, the rate of channel opening and the slower rate of channel desensitization, together with channel deactivation and recovery rates, controls the synaptic excitability. In this regard, a slowly gated AMPA channel bound with a TARP would have a longer rising phase of the excitatory postsynaptic potential (EPSP) and may have a delayed initiation of postsynaptic action potential after EPSP^45^.

The current understanding of the action of TARPs is mostly based on various studies of γ-2, including structural studies as well. To date, TARPs are known to increase in both single-channel conductance and agonist efficacy^15,16^, both of which contribute to potentiation of macroscopic peak current amplitude. Larger whole-cell currents through an AMPA channel bound with auxiliary subunits can be also explained by increased occupancy of high- compared to low-conductance levels. Our study shows that both the channel-opening and the channel-closing rates of an AMPA receptor channels are slowed when TARPs are present, as compared with the same channel but without TARP. A longer open-channel duration, albeit a slower open-channel formation, can lead to a higher volume of ionic flux or a higher charge transfer through the open channel, thereby producing a higher peak current amplitude. In fact, different from γ-2, γ-4 barely changes the affinity towards glutamate (as measured by K_1_ value in Table 1). Consequently, the elevation of a macroscopic current response to ligand of the same concentration (as shown in Fig. 1B) cannot be explained by the dose-response effect or the increase of glutamate affinity alone; rather, such potentiation of the macroscopic current response could be ascribed to the kinetic effect of γ-4 on slowing down the channel-opening rate and more so the channel-closing rate, thus leading to a higher charge transfer.

## Methods

### TARP and receptor expression

The γ-2 and γ-4 plasmids were generously provided by Prof. Susumu Tomita. The flip isoform of GluA4^30^ with and without a TARP was transiently expressed in HEK-293S cell. The tissue culture and transfection procedure were described before^46^. In brief, HEK-293S cells were cultured in Dulbecco’s modified Eagle’s medium supplemented with 10% fetal bovine serum, 100 U/mL penicillin, and 100 μg/mL streptomycin in a 37 °C, 6% CO_2_, humidified incubator. In co-transfecting a TARP, we used 1:1 and 1:2 weight ratio of the plasmid of GluA4 to a TARP (GluA4 plasmid was 3–6 μg/35 mm dish). Co-transfection also included green fluorescent protein (GFP) and large T-antigen (TAg)^46^. We also added a 2,3-benzodiazepine inhibitor^47^ after transfection to prevent cell toxicity due to transfection of TARP. Transfected cells were grown for >48 h before recording.

### Whole-cell current recording and laser-pulse photolysis

The procedure of recording glutamate-induced whole-cell current was described before^46^. Briefly, the pipet solution or internal solution contained (in mM) 110 CsF, 30 CsCl, 4 NaCl, 0.5 CaCl_2_, 5 EGTA, and 10 HEPES (pH 7.4 adjusted by NaOH). The external cellular solution or bath solution contained (in mM) 150 NaCl, 3 KCl, 1 CaCl_2_, 1 MgCl_2_, and 10 HEPES (pH 7.4 adjusted by NaOH)^46^. The GFP fluorescence in transfected cells was visualized on an Axiovert S100 microscope with a fluorescent detection system from Carl Zeiss. The whole-cell current was recorded using an Axopatch-200B amplifier at a cutoff frequency of 2–20 kHz by a built-in, four-pole Bessel filter and digitized at 5–50 kHz sampling frequency using an Axon Digidata 1322 A. Unless otherwise noted, all experiments were performed with transfected HEK-293S cells voltage-clamped at −60 mV and at 22 °C.

The laser-pulse photolysis technique was used to measure the channel-opening kinetic constants of an AMPA receptor, as we previously reported^46^. A patched cell was first equilibrated with a caged glutamate [γ-*O*-(α-carboxy-2-nitrobenzyl) glutamate from Invitrogen] solution for 250 ms prior to laser photolysis to photolytically liberate free glutamate. A Minilite II pulsed Q-switched Nd:YAG laser (Continuum, Santa Clara, CA) delivered single pulses at 355 nm with a pulse length of 8 ns. To determine the concentration of the photolytically released glutamate, we used two free glutamate solutions with known concentration on the same cell before and after a laser pulse. The whole cell current amplitudes of the released glutamate were compared to the amplitudes of the free glutamate, with reference to a dose-response curve^48^. A fast solution flow technique with a rise time of 1 ms (90% current response) was used to deliver free glutamate or the caged glutamate; we used the same technique to measure dose-response and current-voltage (I-V) relationships as well as desensitization rate^48^.

### Data analysis for whole-cell current traces from flow measurements

**Figure 5:**
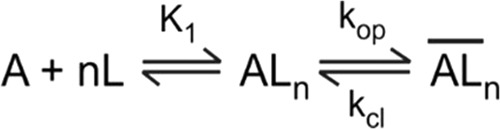
A general, minimal mechanism of channel opening. It is noted that we have previously proposed this mechanism (as in ref.^49^). In brief, A stands for the active, unliganded form of the receptor, L the ligand or glutamate, AL_n_ the closed-channel state with n ligand molecules bound, and $\overline{A{L}_{n}}$ the open-channel state. The number of glutamate molecules to bind to the receptor and to open its channel, n, can be from 1 to 4, assuming that each subunit in a tetrameric complex has one glutamate binding site. It is further assumed that a ligand does not dissociate from the open-channel state. The k_op_ and k_cl_ are the channel-opening and channel-closing rate constants, respectively. Without contrary evidence, it is assumed that glutamate binds with equal affinity or K_1_, the intrinsic equilibrium dissociation constant, at all binding steps.

The analysis of glutamate-induced AMPA receptor current was based on a general, minimal mechanism of channel opening as shown in Fig. 5. Based on this general, minimal mechanism of channel opening, Eq. 1 was derived^48^. In Eq. 1, I_A_ is the observed amplitude of macroscopic current amplitude, I_M_ is the current per mole of receptor, R_M_ the number of moles of receptors on the cell surface, and Φ^−1^ the channel opening equilibrium constant. A non-linear regression was performed to determine K_1_, along with other parameters, from a dose-response relationship. Each of the current traces was corrected for desensitization before it was used in calculating dose-response relationship^48^.

1 $${I}_{A}={I}_{M}{R}_{M}\frac{{L}^{n}}{{L}^{n}+{\rm{\Phi }}{(L+{K}_{1})}^{n}}$$

### Data analysis for channel-opening rate measurement

The k_op_ and k_cl_ values were determined from the rising phase of a whole-cell current trace in a laser-pulse photolysis experiment. These rate constants reflect the ensemble kinetic properties of channels in response to the binding of glutamate. In a laser-pulse photolysis measurement, we observed that the whole-cell current rise followed a single-exponential rate process (>95%) in the entire range of ligand (glutamate) concentrations we were able to measure (70–475 µM glutamate). The observed first-order rate constant of channel opening, k_obs_, was estimated by using Eq. 2

2 $${I}_{t}={I}_{max}(1-{e}^{-{k}_{obs}t})$$

where *I*_*t*_ and *I*_*max*_ represent the whole-cell current amplitude at time *t*, and the maximum current amplitude, respectively. Based on the general mechanism of channel opening, Eq. 3 was derived to describe the relationship between k_obs_ and ligand concentration:

3 $${k}_{obs}={k}_{cl}+{k}_{op}{(\frac{L}{L+{K}_{1}})}^{n}$$

In deriving Eq. 3, we assumed the rate of ligand binding was fast relative to the rate of channel opening. This assumption was consistent with our observation that the whole-cell current rise followed a first-order rate law (Eq. 2) in the entire range of glutamate concentrations not only in this study but also in all of our previous studies of other AMPA receptors^29,30,48–53^.

Unless otherwise noted, each data point shown in a plot is an average of at least three measurements collected from at least three cells; mean ± SEM is reported. Origin was used for both linear regression and nonlinear fitting.

## Supplementary information

Stargazin and γ4 slow the rate of channel opening and closing of GluA4 AMPA receptors
