# Supplementary material for: Stargazin and γ4 slow the channel opening and closing rates of GluA4 AMPA receptors
Source: Sci Rep. 2019 Jul 2;9:9570. doi: 10.1038/s41598-019-45870-0 (PMC6606765; doi:10.1038/s41598-019-45870-0)
Supplement: Supplementary file 1 — Stargazin and γ4 slow the rate of channel opening and closing of GluA4 AMPA receptors [file 41598_2019_45870_MOESM1_ESM.pdf]

# Supporting Information

# Stargazin and γ4 slow the rate of channel opening and closing of GluA4 AMPA receptors*

**Vincen D. Pierce, and Li Niu[1]**

From the Department of Chemistry and Center for Neuroscience Research, University at Albany, SUNY, Albany, New York 12222

---

The supporting information contains various nonlinear fits of the channel opening traces from the laser-pulse photolysis experiments. Here are the summary of the data from non-linear fitting.

(1) The Hill coefficients for the fitting of the dose-response curves of GluA4/γ2 and GluA4/γ4 are provided in Table S1. The corresponding $EC_{50}$ values are provided in Table 1.

(2) $K_1$ value for GluA4/γ2 and GluA4/γ4 are estimated to be 0.4-0.6 mM (Table S2B) and 0.6-0.8 mM (Table S3B). These values are in good agreement with those obtained from dose-response data as shown in Table 1 in the text.

(3) The number of ligand molecules bound per receptor, n, for either channel type is around 2 (Table S2A, S3A and S4). It should be emphasized that the n value can be also obtained from fitting of the dose-response curve. However, the n value is obtained from Eq 1 derived from a general mechanism of channel opening. The n value refers to the number of ligand molecules that are bound to and open the channel. In contrast, the Hill coefficient is obtained from the use of the Hill equation for the analysis of the same dose-response relationship. Yet, the Hill coefficient refers to cooperativity.

(4) When the values of $K_1$ and n are fixed, the use of non-linear regression yielded $k_{cl}$ of 1,200 and 1,100 $s^{-1}$ for GluA4/γ2 and GluA4/γ4 (Table S5); similarly, the best fit of $k_{op}$ is 1.3 X $10^4$ and 1.6 x $10^4$ for GluA4/γ2 and GluA4/γ4, respectively (Table S5). All of these values are in good agreement with the linear fit, as shown in Fig 4 and Table 1 in the text.

**Table S1 –** The Hill coefficients of the dose-response curves of GluA4/γ2 and GluA4/γ4. The corresponding $EC_{50}$ values are provided in Table 1.

| Receptor Types | Hill Coefficient |
|---|---|
| GluA4 | 2.18 ± 0.21 |
| GluA4/γ2 | 1.07 ± 0.14 |
| GluA4/γ4 | 1.08 ± 0.10 |

**Table S2A – Nonlinear fitting of $k_{obs}$ as a function of glutamate concentration for GluA4/γ2, when $k_{cl}$ was fixed at different values.** For the fitting, $k_{cl}$ was fixed at three different values (see the footnote) for estimating $k_{op}$, $K_1$ and n using eq 3 (Experimental Procedures). The rationale for fixing $k_{cl}$ was presented in the text. In short, when $L<<K_1$, eq 3 becomes $k_{obs} \approx k_{cl}$. The $k_{obs}$ value at low glutamate concentration for this channel was about 1,200 $s^{-1}$.

| Initial Values | | | | Outputs | | | |
|---|---|---|---|---|---|---|---|
| $k_{cl}$[a] ($s^{-1}$) | $k_{op}$ ($s^{-1}$) | $K_1$ (mM) | n | $k_{op}$ ($s^{-1}$) | $K_1$ (mM) | n | $R^2$ |
| 1000 | 7000 | 0.5 | 2 | 12416 | 0.50 | 1.64 | 0.89 |
| 1000 | 10000 | 0.6 | 1 | 8630 | 0.64 | 1.19 | 0.83 |
| 1000 | 13000 | 1 | 3 | 32468 | 1.13 | 1.63 | 0.90 |
| 1000 | 16000 | 0.4 | 4 | 14375 | 0.57 | 1.67 | 0.89 |
| 1000 | 19000 | 0.6 | 2 | 20069 | 0.65 | 1.79 | 0.89 |
| 1200 | 7000 | 0.5 | 2 | 19761 | 0.59 | 1.99 | 0.91 |
| 1200 | 10000 | 0.6 | 1 | 8340 | 0.66 | 1.24 | 0.80 |
| 1200 | 13000 | 1 | 3 | 27881 | 1.09 | 1.63 | 0.92 |
| 1200 | 16000 | 0.4 | 4 | 20346 | 0.38 | 2.57 | 0.90 |
| 1200 | 19000 | 0.6 | 2 | 19798 | 0.60 | 1.97 | 0.91 |
| 1400 | 7000 | 0.5 | 2 | 25725 | 0.56 | 2.39 | 0.93 |
| 1400 | 10000 | 0.6 | 1 | 7451 | 0.67 | 1.28 | 0.74 |
| 1400 | 13000 | 1 | 3 | 32354 | 1.04 | 1.86 | 0.92 |
| 1400 | 16000 | 0.4 | 4 | 21615 | 0.39 | 2.81 | 0.93 |
| 1400 | 19000 | 0.6 | 2 | 27535 | 0.66 | 2.23 | 0.93 |

| Final outputs | $k_{cl}$[a] ($s^{-1}$) | $k_{op}$ ($s^{-1}$) | $K_1$ (mM) | n | $R^2$ |
|---|---|---|---|---|---|
| | 1000 | 17591 | 0.70 | 1.59 | 0.878 |
| Ave. | 1200 | 19225 | 0.67 | 1.88 | 0.887 |
| | 1400 | 22936 | 0.66 | 2.11 | 0.890 |

[a]Three fixed values of $k_{cl}$ were chosen: 1000 $s^{-1}$, 1200 $s^{-1}$, and 1800 $s^{-1}$.

**Table S2B – Nonlinear fitting of $k_{obs}$ as a function of glutamate concentration for GluA4/γ2, when $k_{cl}$ and n were fixed at different values.** The fitting was similar to those in Table S1A except that n, the number of ligand molecules that are bound to the receptor and the open the channel was also mixed at 2. The choice of 2 was based on the fitting in Table S1A, where the average of n from various fits is about 2. Furthermore, we ruled out the fractional numbers as meaningful n value in our fitting.

| Initial Values | | | | Outputs | | |
|---|---|---|---|---|---|---|
| $k_{cl}$[a] ($s^{-1}$) | $k_{op}$ ($s^{-1}$) | $K_1$ (mM) | n[b] | $k_{op}$ ($s^{-1}$) | $K_1$ (mM) | $R^2$ |
| 1000 | 7000 | 0.5 | 2 | 9778 | 0.29 | 0.88 |
| 1000 | 10000 | 0.6 | 2 | 12938 | 0.38 | 0.88 |
| 1000 | 13000 | 1 | 2 | 18525 | 0.50 | 0.88 |
| 1000 | 16000 | 0.4 | 2 | 15773 | 0.45 | 0.88 |
| 1000 | 19000 | 0.6 | 2 | 20425 | 0.54 | 0.88 |
| 1200 | 7000 | 0.5 | 2 | 11121 | 0.37 | 0.89 |
| 1200 | 10000 | 0.6 | 2 | 13999 | 0.45 | 0.90 |
| 1200 | 13000 | 1 | 2 | 18566 | 0.55 | 0.91 |
| 1200 | 16000 | 0.4 | 2 | 14400 | 0.45 | 0.90 |
| 1200 | 19000 | 0.6 | 2 | 19950 | 0.57 | 0.91 |
| 1400 | 7000 | 0.5 | 2 | 18987 | 0.63 | 0.91 |
| 1400 | 10000 | 0.6 | 2 | 17911 | 0.61 | 0.91 |
| 1400 | 13000 | 1 | 2 | 17900 | 0.60 | 0.91 |
| 1400 | 16000 | 0.4 | 2 | 21008 | 0.67 | 0.91 |
| 1400 | 19000 | 0.6 | 2 | 19000 | 0.63 | 0.91 |

| Final Outputs | $k_{cl}$[a] ($s^{-1}$) | n[b] | $k_{op}$ ($s^{-1}$) | $K_1$ (mM) | $R^2$ |
|---|---|---|---|---|---|
| | 1000 | 2 | 15488 | 0.43 | 0.88 |
| Ave. | 1200 | 2 | 15607 | 0.48 | 0.90 |
| | 1400 | 2 | 18961 | 0.63 | 0.91 |

[a] Three fixed values of $k_{cl}$ were chosen: 1000 $s^{-1}$, 1200 $s^{-1}$, and 1800 $s^{-1}$.
[b] The fixed value of n is determined from the results in Table 1A.

**Table S3A – Nonlinear fitting of $k_{obs}$ as a function of glutamate concentration for GluA4/γ4, when $k_{cl}$ was fixed at different values.** The fitting routine was similar to the one as in Table S1A. The only exception is that the fitting we did below was for GluA4/γ4 channels.

| Initial values | | | | Outputs | | | |
|---|---|---|---|---|---|---|---|
| $k_{cl}$[a] ($s^{-1}$) | $k_{op}$ ($s^{-1}$) | $K_1$ (mM) | n | $k_{op}$ ($s^{-1}$) | $K_1$ (mM) | n | $R^2$ |
| 900 | 11000 | 0.5 | 2 | 11140 | 0.53 | 1.96 | 0.84 |
| 900 | 14000 | 0.6 | 1 | 7431 | 0.73 | 1.37 | 0.92 |
| 900 | 17000 | 1 | 3 | 21807 | 1.13 | 1.69 | 0.84 |
| 900 | 20000 | 0.4 | 4 | 23617 | 0.45 | 2.77 | 0.56 |
| 900 | 23000 | 0.6 | 2 | 20317 | 0.64 | 2.17 | 0.73 |
| 1100 | 11000 | 0.5 | 2 | 10326 | 0.52 | 2.12 | 0.91 |
| 1100 | 14000 | 0.6 | 1 | 7631 | 0.79 | 1.53 | 0.89 |
| 1100 | 17000 | 1 | 3 | 20937 | 1.09 | 1.85 | 0.90 |
| 1100 | 20000 | 0.4 | 4 | 23672 | 0.41 | 3.23 | 0.79 |
| 1100 | 23000 | 0.6 | 2 | 19310 | 0.68 | 2.27 | 0.88 |
| 1300 | 11000 | 0.5 | 2 | 12384 | 0.58 | 2.41 | 0.84 |
| 1300 | 14000 | 0.6 | 1 | 5780 | 0.77 | 1.59 | 0.75 |
| 1300 | 17000 | 1 | 3 | 20180 | 1.08 | 2.02 | 0.83 |
| 1300 | 20000 | 0.4 | 4 | 22431 | 0.40 | 3.53 | 0.86 |
| 1300 | 23000 | 0.6 | 2 | 17424 | 0.66 | 2.50 | 0.85 |

| Final Outputs | $k_{cl}$[a] ($s^{-1}$) | $k_{op}$ ($s^{-1}$) | $K_1$ (mM) | n | $R^2$ |
|---|---|---|---|---|---|
| | 900 | 16862 | 0.70 | 1.99 | 0.78 |
| Ave. | 1100 | 16375 | 0.70 | 2.20 | 0.88 |
| | 1300 | 15640 | 0.70 | 2.41 | 0.83 |

[a]Three fixed values of $k_{cl}$ were chosen: 9,00 $s^{-1}$, 1,100 $s^{-1}$, and 1,300 $s^{-1}$.

**Table S3B – Nonlinear fitting of $k_{obs}$ as a function of glutamate concentration for GluA4/γ4, when $k_{cl}$ and n were fixed at different values.** The fitting was similar to Table S1B, except that the fitting was done for the GluA4/γ4 data. The choice of n was based on the fitted data from Table S2A. We set n to be integer as well.

| Initial Values | | | | Outputs | | |
|---|---|---|---|---|---|---|
| $k_{cl}$[a] ($s^{-1}$) | $k_{op}$ ($s^{-1}$) | $K_1$ (mM) | n[b] | $k_{op}$ ($s^{-1}$) | $K_1$ (mM) | $R^2$ |
| 900 | 11000 | 0.5 | 2 | 11000 | 0.50 | 0.83 |
| 900 | 14000 | 0.6 | 2 | 14000 | 0.60 | 0.81 |
| 900 | 17000 | 1 | 2 | 20719 | 0.78 | 0.77 |
| 900 | 20000 | 0.4 | 2 | 12359 | 0.54 | 0.82 |
| 900 | 23000 | 0.6 | 2 | 17681 | 0.71 | 0.78 |
| 1100 | 11000 | 0.5 | 2 | 9900 | 0.56 | 0.91 |
| 1100 | 14000 | 0.6 | 2 | 13463 | 0.69 | 0.91 |
| 1100 | 17000 | 1 | 2 | 19231 | 0.88 | 0.90 |
| 1100 | 20000 | 0.4 | 2 | 10018 | 0.56 | 0.91 |
| 1100 | 23000 | 0.6 | 2 | 15597 | 0.76 | 0.90 |
| 1300 | 11000 | 0.5 | 2 | 8078 | 0.64 | 0.81 |
| 1300 | 14000 | 0.6 | 2 | 11025 | 0.76 | 0.82 |
| 1300 | 17000 | 1 | 2 | 17000 | 1.00 | 0.83 |
| 1300 | 20000 | 0.4 | 2 | 8456 | 0.64 | 0.81 |
| 1300 | 23000 | 0.6 | 2 | 13225 | 0.84 | 0.82 |

| Final Outputs | $k_{cl}$[a] ($s^{-1}$) | $k_{op}$ ($s^{-1}$) | $K_1$ (mM) | n[b] | $R^2$ |
|---|---|---|---|---|---|
| | 900 | 15152 | 0.63 | 2 | 0.80 |
| Ave. | 1100 | 13642 | 0.69 | 2 | 0.91 |
| | 1300 | 11557 | 0.78 | 2 | 0.82 |
| | | | | | |

[a]Three fixed values of $k_{cl}$ were chosen: 900 $s^{-1}$, 1100 $s^{-1}$, and 1300 $s^{-1}$.
[b]The fixed value of n is determined from the results in Table 2A.

**Table S4 – Non-linear fitting of $k_{obs}$ with a fixed $k_{cl}$ and $K_1$ for estimating $k_{op}$ and n for GluA4/γ2 and GluA4/γ4 channels.** To achieve a better estimate of n and $k_{op}$ by non-linear regression (Simplex algorithm) using eq 3, the values of $k_{cl}$ were fixed. According to equation 3, $k_{obs} \approx k_{cl}$ when $L \ll K1$, suggesting that (a) $k_{obs}$ at a low glutamate concentration would reflect $k_{cl}$ and (b) the value of $k_{cl}$ is independent variable. Based on this rationale, we identified a $k_{obs}$ determined at ~4% fraction of the open channel to be the $k_{cl}$ for each channel combination (e.g. $k_{cl}$ = 1,200 $s^{-1}$ for GluA4/γ2). In addition, $K_1$ value was also fixed at 0.42 mM for GluA4/γ2 and 0.77 mM for GluA4/γ4. These values were obtained from analysis of the dose-response data.

| Receptor | $K_1$[a] (mM) | $k_{cl}$[b] ($s^{-1}$) | $k_{op}$ ($s^{-1}$) | n | $R^2$ |
|---|---|---|---|---|---|
| **$GluA4_{flip}$/γ2** | 0.42 | $1.2 \times 10^3$ | $(1.30 \pm 0.27) \times 10^4$ | 2.00 ± 0.20 | 0.90 |
| **$GluA4_{flip}$/γ4** | 0.77 | $1.1 \times 10^3$ | $(1.71 \pm 0.54) \times 10^4$ | 2.06 ± 0.21 | 0.90 |

[a, b] values were fixed for the non-linear fitting.

**Table S5 – Non-linear fitting of $k_{obs}$ with a fixed $K_1$ and n for GluA4/γ2 and GluA4/γ4 channels**

| Receptor | $K_1$[a] (mM) | n[b] | $k_{cl}$ ($s^{-1}$) | $k_{op}$ ($s^{-1}$) | $R^2$ |
|---|---|---|---|---|---|
| **GluA4$_{flip}$/γ2** | 0.42 | 2 | $(1.20 \pm 0.11) \times 10^3$ | $(1.30 \pm 0.12) \times 10^4$ | 0.90 |
| **GluA4$_{flip}$/γ4** | 0.77 | 2 | $(1.10 \pm 0.04) \times 10^3$ | $(1.57 \pm 0.12) \times 10^4$ | 0.90 |

[a, b] values are fixed for the non-linear fitting.
